# The Genetic Architecture of Nitrogen Use Efficiency in Switchgrass (*Panicum virgatum* L.)

**DOI:** 10.3389/fpls.2022.893610

**Published:** 2022-05-02

**Authors:** Vivek Shrestha, Hari B. Chhetri, David Kainer, Yaping Xu, Lance Hamilton, Cristiano Piasecki, Ben Wolfe, Xueyan Wang, Malay Saha, Daniel Jacobson, Reginald J. Millwood, Mitra Mazarei, C. Neal Stewart

**Affiliations:** ^1^Department of Plant Sciences, The University of Tennessee, Knoxville, Knoxville, TN, United States; ^2^Center for Bioenergy Innovation, Oak Ridge National Laboratory, Oak Ridge, TN, United States; ^3^ATSI Brazil Pesquisa e Consultoria, Passo Fundo, Brazil; ^4^Noble Research Institute, Ardmore, OK, United States

**Keywords:** nitrogen use efficiency, nitrogen remobilization efficiency, switchgrass, accessions, genome wide association study

## Abstract

Switchgrass (*Panicum virgatum* L.) has immense potential as a bioenergy crop with the aim of producing biofuel as an end goal. Nitrogen (N)-related sustainability traits, such as nitrogen use efficiency (NUE) and nitrogen remobilization efficiency (NRE), are important factors affecting switchgrass quality and productivity. Hence, it is imperative to develop nitrogen use-efficient switchgrass accessions by exploring the genetic basis of NUE in switchgrass. For that, we used 331 diverse field-grown switchgrass accessions planted under low and moderate N fertility treatments. We performed a genome wide association study (GWAS) in a holistic manner where we not only considered NUE as a single trait but also used its related phenotypic traits, such as total dry biomass at low N and moderate N, and nitrogen use index, such as NRE. We have evaluated the phenotypic characterization of the NUE and the related traits, highlighted their relationship using correlation analysis, and identified the top ten nitrogen use-efficient switchgrass accessions. Our GWAS analysis identified 19 unique single nucleotide polymorphisms (SNPs) and 32 candidate genes. Two promising GWAS candidate genes, *caffeoyl-CoA O-methyltransferase* (*CCoAOMT*) and *alfin-like 6* (*AL6*), were further supported by linkage disequilibrium (LD) analysis. Finally, we discussed the potential role of nitrogen in modulating the expression of these two genes. Our findings have opened avenues for the development of improved nitrogen use-efficient switchgrass lines.

## Introduction

Nitrogen (N) is a major macronutrient, which is essential for plant biomass and yield production. In the past 50 years, the application of synthetic N fertilizer to farmland has resulted in a dramatic increase in crop yields but with considerable negative impacts on the environment (Han et al., 2015). A large proportion of the applied N (50–70%) is lost from the plant-soil framework (Lam et al., 1996; Ranjan and Yadav, 2019). Excessive use of N fertilizer degrades the natural resources, such as air, soil, underground water, and contributes to global warming (Byrnes, 1990; Glass et al., 2002; Chien et al., 2016). Synthetic nitrogen fertilizer production and N_2_O from use of synthetic N fertilizers contribute to 0.8 and 1.3% of global greenhouse gas emissions, respectively (Jensen and Schjoerring, 2011; Langholtz et al., 2021). Therefore, new solutions are needed to decrease applied N without yield penalty to maximize the nitrogen use efficiency (NUE) of crops. A study conducted on simulating 20% increase in NUE in row crops has shown to reduce N requirements by 1.27 metric tons per year and increase farmers’ net profits by 1.6% per year by 2026 over the base simulation for the same period (Langholtz et al., 2021).

Switchgrass (*Panicum virgatum* L.) is a perennial grass native to North America and developed as a potential bioenergy crop due to its high biomass production, ability to grow in marginal land, low input requirements for maintenance, and high cellulose content (Sanderson et al., 1996; Adler et al., 2006). It has been reported that a substantial proportion of nutritive elements are removed with each biomass harvest from switchgrass, although it remobilizes nutrients from shoots to roots, each growing cycle during senescence (Yang et al., 2009). Studies have shown that the total N removed with one-cut fall biomass harvest ranges from 31 to 63 kg N per ha per year, while, for two-cut system, it ranges from 90 to 144 kg per ha per year over the 5 years of measurement (Reynolds et al., 2000). With such high nutrient withdrawal, it is inevitable that N depletion from soil will occur over time and necessitate the addition of N fertilizer to maintain sustainable switchgrass production. Therefore, the development of nitrogen-use-efficient switchgrass cultivars is imperative for the sustainable production of biofuel. To develop nitrogen-use-efficient switchgrass cultivars, we need to have a better understanding of the NUE and its genetic architecture.

Several definitions and calculations of NUE have been published, which encompass a wide range of NUE calculations, as well as acknowledge that different NUE indices have distinctive functions (Good et al., 2004; Ladha et al., 2005; Dobermann, 2007; Sadeghpour et al., 2014; Ernst et al., 2020; Congreves et al., 2021). Hence, it is recommended to use multiple NUE approaches or NUE-related indices to ensure the better representation of different insights (Van, 2007; Fixen et al., 2015; Congreves et al., 2021). NUE is complex and possesses several components. Nitrogen remobilization efficiency (NRE) is one of the important components of NUE in switchgrass. For perennial grass, such as switchgrass, improving NRE from aboveground to underground organs during yearly shoot senescence is equally important for sustainable production of switchgrass (Yang et al., 2016) and should be considered for developing nitrogen use-efficient switchgrass varieties. NRE has been investigated intensively at the agronomic level (Lemus et al., 2008; Yang et al., 2009; Strullu et al., 2011; Dohleman et al., 2012; o Di Nasso et al., 2013) but very limited at the genetic level. Therefore, the present study will add a foundational understanding of the genetic basis of NRE in switchgrass.

Nitrogen use efficiency is a quantitative trait and governed by polygenes. A quantitative genetics approach, such as genome wide association study (GWAS), has been a powerful tool to dissect the genetic architecture of complex traits (Lander and Schork, 1994; Mackay, 2001; Doerge, 2002). GWAS for NUE has been used in several crops, such as barley (Karunarathne et al., 2020), maize (Morosini et al., 2017; He et al., 2020), rice (Xin et al., 2021), wheat (Cormier et al., 2014; Hitz et al., 2017), and mustard (Gupta et al., 2021). Several genes were highlighted in the previous studies, regulating the genetic basis of NUE. Previous studies showed ammonium (AMT) and nitrate transporters (NRT1/NRT2) play important roles in the N uptake and transport in rice (Huang et al., 2017; Wang et al., 2018) and barley (Karunarathne et al., 2020). Several transcription factors and protein kinases were reported in a plant N regulatory network of *Brassicajuncea* (Goel et al., 2018). Studies also reported on manipulation of genes, regulating primary and secondary N assimilatory pathways to improve NUE (Pathak et al., 2008; Karunarathne et al., 2020). GWAS and downstream genomic analysis was found to be effective in understanding the genetic basis of NUE in several of these crops, but the genetic basis of NUE in switchgrass is not studied yet.

It has been reported that the most fundamental approach to enhanced NUE cultivar development necessitates plant evaluation under both low and high N conditions (Han et al., 2015). This helps in comparative evaluation of performance of a genotype at both low and high N conditions and facilitates the identification of the NUE-efficient genotype (Han et al., 2015). In the present study, our switchgrass experimental population is also grown at a contrasting N fertility condition—low N and moderate N conditions. Since NUE is regulated by biological, physiological, environmental, genetic, agronomic, and developmental factors (Congreves et al., 2021), no single measure of NUE could unravel the complexity of its genetic basis. Therefore, in the present study, we have used a holistic approach to target the genetic basis of NUE by using GWAS of not only the absolute dry biomass trait at low and moderate N but also the different NUE indices, such as NUE and NRE. We performed GWAS analysis on total dry biomass and NUE using 331 diverse switchgrass accessions as well as NRE using 150 diverse accessions that were field grown under two different N treatments. These traits are closely related to NUE in plants and have been widely used in the physiological, agronomical, and genetic studies of NUE (Ball Coelho et al., 2006; Lemus et al., 2008; Yang et al., 2009; Sadeghpour et al., 2014; Karunarathne et al., 2020). Our assumption is that the genes and the biological process uncovered from the GWAS analysis of the NUE and related traits should depict the genetic basis to improve the NUE. Here, we highlighted the several SNPs and candidate genes for NUE and related traits in switchgrass. We used linkage disequilibrium analysis to further support the GWAS-derived candidate genes and, finally, put forward an interesting discussion on the role of N in modulating the expression of these GWAS candidate genes. To our knowledge, this is the first report on the genetic basis of NUE of field-grown switchgrass.

## Materials and Methods

### Field Experimental Design

A highly diverse panel of 331 switchgrass accessions (Lovell et al., 2021) was planted in May 2019 in a 75.2 m × 122.5 m dimension at The University of Tennessee Plant Sciences Unit of the East Tennessee Research and Education Center (ETREC) (latitude: 35°54′11.14″N; longitude: 83°57′33.31″W; and elevation: 255.7 m.). Planting details and experimental design can be found in Li et al. (2020) and Xu et al. (2021). Briefly, the 331 accessions were planted under two nitrogen (N) fertility treatments: one with moderate nitrogen (135 kg of N ha^–1^), while the other with low (0 kg of N ha^–1^) supplementation in 2019 and 2020. Each of the 330 accessions has four replicates in the field (2 replicates per N treatment), totaling 1,320 switchgrass plants, plusone control AP13 (“Alamo”) grown with 40 replicates (20 at low and 20 at moderate nitrogen), which were arranged in honeycomb design with ∼2.5 m interplant spacing. AP13 is the reference sequenced lowland cultivar, which is broadly used as a reference genome for switchgrass (Lovell et al., 2021). The field is equipped with a weather station (HOBO, RX3000, Bourne, MA, United States). The average temperature was 23.8°C, and the average precipitation was 0.03 mm during the period of July 2020 to December 2020.

### Biomass Quantification

The dry biomass for each switchgrass accession was quantified as previously described (Li et al., 2020). Briefly, the biomass for each plant was quantified at the end of season after plant senescence. The aboveground biomass of individual plants was harvested and weighed. Subsequently, ten random tillers were harvested from each plant and oven-dried at 45°C for 72 h. Weight of the 10 tillers before and after drying was used to determine the ratio of dry-to-fresh weight. Total dry biomass was determined by calculating the percentage of water loss recorded for each subsample and subsequently applying the water loss percentage to the respective total wet biomass weight for individual plants.

### Nitrogen Use Efficiency Index Calculation

The following equations were adopted from Ball Coelho et al. (2006), Lemus et al. (2008), Sadeghpour et al. (2014) to calculate NUE.


(1) $$N\,U\,E\,\left(K\,g\,K\,g/m^{2}\right)=\left(B\,M\,Y_{f}-B\,M\,Y_{u}\right)/N_{s}$$

BMY_f_ = Biomass yield (BMY) of the fertilized plant.

BMY_u_ = Biomass yield of the unfertilized plant.

N_s_ = Nitrogen fertilizer at the given rate.

### Nitrogen Quantification

The detail of the nitrogen quantification was previously described in Xu et al. (2021). Briefly, nitrogen content of the aboveground biomass was measured *via* near-infrared spectroscopy (NIRS) using a FOSS 6500 NIR system (Silver Spring, MD, United States). Nitrogen content was measured at two developmental time points during the field-growing season: one in August 2020 at mid-season (M) and the other in December 2020 at the end of season (S). A total of 150 accessions in two replicates in two N treatments and two time points (150 × 2 × 2 × 2 = 1,200 samples) were used, i.e., 300 plants at mid-season at low N, 300 plants at mid-season at moderate N, 300 plants at the end of season at low N, and 300 plants at the end of season at moderate N were used. The 150 accessions were chosen based on biomass yield data of the 2019 growing season (Li et al., 2020). Two tillers containing both stems and leaves were collected from each plant, and the samples were oven dried at 45°C for 72 h. The dried tillers were then chipped into 5–8 pieces, each around 4–6 inch long, prior to milling. The chipped samples were milled with a Wiley Mill (Thomas Scientific, Model 4, Swedesboro, NJ, United States) through a 20-mesh screen (1.0-mm particle size).

### Nitrogen Remobilization Efficiency Index Calculation

Nitrogen remobilization efficiency index calculation was performed by adopting the equation from Yang et al. (2009).


(2) N⁢R⁢E=(M-S)/M

where *M* and *S* represent nitrogen content at the green/mature (mid-season) and senescent (the end of season) stages, respectively. Since we took N samples at two different time points (M and S) from each of the 150 accessions grown at low N condition and moderate N condition, we calculated NRE for low N and NRE for moderate N condition separately.

### Phenotypic Data

We evaluated five NUE-related traits. All traits were treated independently. NUE ratio/indices traits were derived prior to calculation of the best linear unbiased predictions (BLUP) to minimize noise. We removed outliers using the median absolute deviation (MAD) method such that any phenotypic values with MAD > 4 from the population median for a particular trait were removed. Variance components were estimated from a mixed linear model where accession and replication were fitted as random effects and were used to estimate broad sense heritability on a line-mean basis as previously described (Holland et al., 2003).

### Genome Wide Association Study and Linkage Disequilibrium Analysis

Methods for SNP variant calling were described previously (Lovell et al., 2021). Briefly, whole-genome resequencing data for 331 genotypes were obtained using Illumina genetic analyzers at the DOE Joint Genome Institute. After removing SNPs with more than 10% missing genotypes, the genotypes with more than 10% missing SNPs, the SNPs with severe departure from Hardy Weinberg Equilibrium (SNPs with HWE > 1E-50 removed), and the SNPs with minor allele frequency (MAF < 0.01), the SNPs with r^2^ ≥ 0.95, a total of 11,976,627 SNPs were available for the downstream analysis.

Genome-wide association tests for all phenotypic traits were performed using the genome-wide complex trait analysis (GCTA) software using the following linear model (Yang et al., 2011). Phenotypic BLUPs, a genetic relationship matrix, and 11,976,627 SNPs were used for the association test. Univariate GWAS was run for six phenotypic traits using the following model:


(3) y=X⁢β+W⁢u+α+ε

where y is a phenotypic vector of size n x 1, with n representing sample size, β is a q x 1 vector of fixed effect that includes the first three eigenvectors from the PCA analysis of the genomic data with its incidence matrix X (an n x q matrix of covariates), u is the p x 1 vector of additive SNP effects with its incidence matrix W (an n x p genotype matrix), α is the n x 1 vector of random effects that include the genetic relationship matrix (GRM), and ε is the n x 1 vector of residual random effects.

Since the Bonferroni threshold for multiple hypothesis correction is too stringent for significant SNPs, the genome-wide threshold of significance in this study was set to 8. × 10^–8^ (1/N, with *N* = 11,976,627 SNPs). Bonferroni assumes each tested SNP is independent, but the presence of LD between SNPs makes that assumption incorrect and overly stringent. Previously, a similar threshold set was being widely used in other species, such as maize and Arabidopsis (Wen et al., 2014; Wu et al., 2021; Zhu et al., 2021). Candidate gene lists were obtained from the 10-kb interval of the peak GWAS SNP. However, if the gene was not found within that range, we chose the nearest gene left and right from the peak GWAS SNPs. Gene annotations of the candidate genes were based on the orthologs of *Arabidopsis thaliana* and rice (*Oryza sativa*).

Pairwise LD values between peak GWAS SNPs and SNPs within 20-kb interval SNPs (10 kb up and downstream) of peak GWAS SNPs were calculated using squared allele-frequency correlations (r^2^) using plink version 1.9 (Chang et al., 2015). All SNPs were filtered at a 1% minor allele frequency. We also computed the proportion of variance in phenotype explained (PVE), as previously described in Shim et al. (2015), for those GWAS peak SNPs that were in moderate-to-strong LD with the SNPs underlying associated candidate genes.

## Results

### Characterization of the Phenotypic Variability of Nitrogen Use Efficiency and the Related Traits

The extent of the phenotypic variability of dry biomass at low and moderate N and NUE was assessed using the 331-switchgrass diversity panel, while the N content at mid-season and the end of season, NRE at low N and NRE at moderate N were assessed using 150 switchgrass accessions grown at low and moderate N conditions. The full data used to perform the phenotypic variability of NUE and the related traits can be found in Supplementary Tables 1, 2. A descriptive statistical summary of the absolute traits (dry biomass at low and moderate N, and N content at mid-season and the end of season), as well as derived NUE indices (NUE and NRE), is presented in Table 1.

**TABLE 1:** A descriptive statistical summary [mean, standard error (SE), and range] of NUE and related traits and their estimated heritability at low- and moderate-N conditions on a switchgrass diversity panel.

Treatment	Trait	Unit	Trait category	No. lines	Mean	SE	Range	Heritability
Low N	Dry biomass end-of-season	Kg	Absolute	676	2.27	0.06	0.08–7.77	0.94
Moderate N	Dry biomass end-of-season	Kg	Absolute	674	2.29	0.06	0.03–9.11	0.93
	NUE	KgKg/m^2^	Derived	670	0.67	1.45	(−242.36)–178.51	0.39
	Midseason nitrogen	%	Absolute	295	1.04	0.01	0.48–1.65	0.59
Low N	End-of-season nitrogen	%	Absolute	297	0.72	0.01	0.26–1.63	0.74
	NRE	%	Derived	294	0.29	0.01	(−1.24)–0.74	0.54
	Midseason nitrogen	%	Absolute	293	1.14	0.01	0.62–1.74	0.54
Moderate N	End-of-season nitrogen	%	Absolute	256	0.76	0.01	0.09–1.50	0.78
	NRE	%	Derived	252	0.32	0.02	(−1.06)–0.92	0.64

*The traits were categorized as absolute and derived traits. The derived traits, such as nitrogen use efficiency (NUE), were calculated from the absolute dry biomass at low and moderate N, while NRE was derived from N content on tillers at midseason and end-of-season growth stages. More details of calculation of these derived traits can be found in Section “Materials and Methods.” Dry biomass was measured in a full panel (330 accessions), with two replications each in low- and moderate-N treatments plus AP13 as control with 20 replications in low N and 20 replications in moderate N, while N content in tillers was measured using 150 accessions, with two replications each of low- and moderate-N treatments.*

We found that the mean dry biomass at the end of season for both low and moderate N conditions in the switchgrass GWAS panel to be similar; however, the range for dry biomass at moderate N (0.03–9.11 Kg) was larger compared to dry biomass at low N (0.08–7.77 Kg) (Table 1). The heritability of dry biomass at both low and moderate N is high (low-0.94 and moderate-0.93) (Table 1), indicating the trait is highly heritable and, therefore, provides the basis for further genetic improvement.

Using the absolute dry biomass traits at low and moderate N, we also calculated NUE as derived traits using Equation 1 (see Section “Materials and Methods”). The mean NUE was found to be 0.67 kgkg/m^2^. We found a broad range of NUE in our diversity panel, including negative values. The negative values indicate that the dry biomass at low N is higher compared to the dry biomass at moderate N, and, as such, those lines could be even more effective to explore further as nitrogen use efficient lines. While the heritability of absolute dry biomass at low and moderate traits was found to be high, the heritability of NUE was found to be low (0.39), indicating derived indices, such as NUE, are much more complex and should be influenced much more by the environment and governed by multiple genes (Table 1).

Regarding the N content in tillers, we found that the mean N content of the switchgrass accessions at the end of season for both low and moderate N conditions (0.72 and 0.76%, respectively) is lower than the mean N content at the mid-season (1.04 and 1.14%, respectively) (Table 1), indicating the plant during senescence lowers the N content in tillers regardless of N fertilizers treatment. Also, the mean N content at moderate N condition was found to be relatively higher than the mean N content at low N at both mid-season and end-of-season harvests (Table 1). Overall, the heritability of the N content for the end of season was found to be higher as compared to mid-season N content; however, the heritability was found to be consistent for mid-season low and moderate N conditions (0.59 for low N and 0.54 for moderate N), as well as the end-of-season low and moderate N conditions (0.74 for low N and 0.78 for moderate N) (Table 1). In addition to the absolute N content in tillers at mid-season and the end of season, we also calculated NRE as a derived trait at low N and moderate N conditions. The mean NRE for moderate N (0.32) was found to be higher compared to mean NRE for low N (0.29), indicating that moderate nitrogen treatment has potentially enhanced the remobilization use efficiency compared to low-nitrogen treatment. The heritability of NRE at moderate N (0.64) is higher than at low N (0.54) (Table 1).

### Top 10 Nitrogen Use Efficiency Accessions in the Switchgrass Genome Wide Association Study Panel

We used two different perspectives to identify the top 10 NUE lines (Table 2); (1) accessions ranked based on the NUE negative value (highest fold change of low N/moderate nitrogen) and (2) accessions ranked based on the NUE positive value (highest fold change of moderate N/low nitrogen). Category 1 indicates those accessions that were highly N use efficient even under the low-N condition, while Category 2 indicates those accessions that showed highest performance when supplemented with moderate N but do poor with low-N treatment. Our analysis identified J504.C as the most efficient NUE accession in Category 1 with fold change of (10.28), followed by J612.C (3.79), J008.C (3.66), Performer TCL-32 (3.09), and J226.A (2.48) (Table 2.1). J504.C, J612.C, J008.C, and J226.A are all lowland tetraploid accessions collected from Missisipppi, Rhode Island, Arkansas, and Texas, United States, respectively. Performer TCL-32 was collected from North Caroline, NC, United States, but the information on its ploidy level was not found.

**TABLE 2:** Top 10 nitrogen use-efficient switchgrass accessions.

2.1	Switchgrass accession	Mean total dry Biomass_Moderate N (Kg)	Mean total dry Biomass_Low N (Kg)	NUE (KgKg/m^2^_)_	Fold change (Low N/Moderate N)
	J504.C	0.62	6.37	−195.61	10.28
	J612.C	0.17	0.66	−16.57	3.79
	J008.C	0.90	3.31	−81.74	3.66
	Performer TCL-32	0.88	2.72	−62.63	3.09
	J226.A	2.26	5.61	−113.70	2.48

**2.2**	**Switchgrass accession**	**Mean total dry Biomass_Moderate N (Kg)**	**Mean total dry biomass_Low N (Kg)**	**NUE (KgKg/m^2^)**	**Fold change (Moderate N/Low N)**

	J477.B	3.24	0.70	86.34	4.61
	J500.B	2.24	0.52	58.52	4.33
	J006.C	1.80	0.52	43.72	3.49
	J466.B	5.42	1.97	117.31	2.75
	J516.C	2.51	0.95	53.08	2.65

*The top 10 accessions were categorized into two categories. 2.1 shows the top five NUE accessions based on the fold change (low N/moderate N), while 2.2 shows the top five NUE accessions based on the fold change (moderate N/low N). The essence of these two categories is mentioned in text. The means from the two replicates from each of low-N and moderate-N accessions were taken from untransformed data to calculate the NUE. The NUE was calculated based on the Equation 1 in Section “Materials and Methods”.*

In Category 2, Accession J477.B was found to be the most efficient NUE accession with fold change of 4.61, followed by J500.B (4.33), J006.C (3.49), J466.B (2.75), and J516.C (2.65) (Table 2.2). J500.B, J006.C, and J516.C are all lowland tetraploid accessions collected from Mississippi, North Carolina, and New York, United States, respectively. J477.B is tetraploid and collected from Arkansas, United States, but the information on lowland or upland was not found. Similar information was not found for Accession J466.B. All the NUE-ranked accessions from the switchgrass GWAS panel can be found in Supplementary Table 3.

### Correlation Analysis Among the Nitrogen Use Efficiency and the Related Traits

**FIGURE 1:**
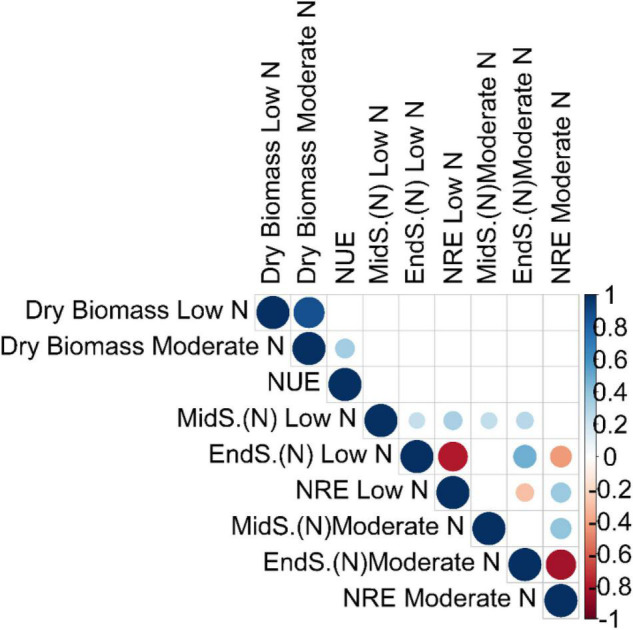
Correlation analysis among the NUE and the related traits. Pairwise Pearson correlation analysis between NUE and related traits was performed using the best linear unbiased predictions (BLUPs) of the switchgrass diversity panel. The correlation matrix was visualized in R v.3.4.3 (R Core Team). Each dot represents a significant correlation coefficient (r) at false discovery rate (qFDR) values < 0.05. Blue dots indicate a strong positive correlation, while light blue indicates a moderate positive correlation. Similarly, red dots indicate a strong negative correlation, while orange indicates a moderate negative correlation. The stronger the correlation (positive or negative), the bigger are the blue and red dots, respectively, and *vice versa*. MidS.(N) and EndS.(N) indicate the % of N content in tillers at the mid season and the end of season, respectively. The unit of dry biomass is Kg; NUE (nitrogen use efficiency) is Kg Kg/m2; and NRE (nitrogen remobilization efficiency) is % (percent of total).

We performed a pairwise Pearson correlation analysis to access the relationship between NUE and the related traits. The pairwise correlation that was significant at false discovery rate (*qFDR*-*values* < 0.05) was only included for further explanation (Figure 1 and Supplementary Tables 4A,B). Dry biomass at low N had a strong positive correlation with dry biomass at moderate N (*r*, 0.88) (Figure 1 and Supplementary Tables 4A,B). NUE had a significant positive correlation with dry biomass at moderate N (*r*, 0.35; *qFDR-value*, 0.00013), while an insignificant negative correlation with dry biomass at low N (*r*, −0.14; *qFDR-value*, 0.22), inferring strong contribution from moderate N toward the plant biomass (Figure 1 and Supplementary Tables 4A,B).

It is surprising to not see any significant correlation between any of the absolute dry biomass traits or their derivative traits (NUE) with that of absolute N content at mid-season and at the end of season, as well as their derivative traits (NRE) (Figure 1 and Supplementary Tables 4A,B). This might be due to the low sample size of N content at mid-season and the end of season at both low and moderate N conditions. It is interesting to observe that NRE is negatively correlated with the end of season (N) while positively correlated with mid-season (N) regardless of two N treatment conditions (Figure 1 and Supplementary Tables 4A,B). The strongest significant negative correlation was found between NRE at moderate N and end-of-season N content at moderate N (*r*, −0.83) (Figure 1 and Supplementary Tables 4A,B)

### Potential Candidate Genes in the Switchgrass Genome Wide Association Study Panel

**FIGURE 2:**
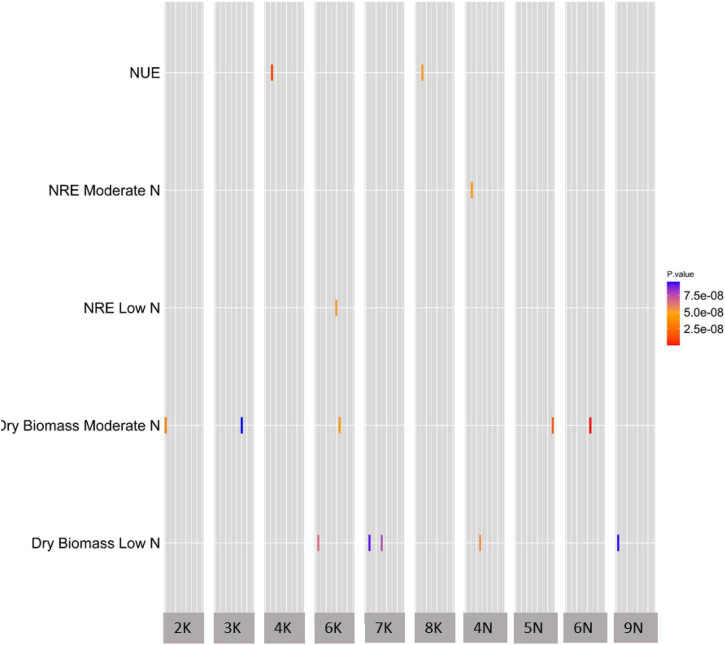
A summary of GWAS of NUE and the related traits. The heatmap of GWAS of NUE and the related traits demonstrating significant SNP distribution across chromosomes. All five traits on y-axis were significant at the genome-wide threshold level of significance at 8.0 × 10^–8^ (1/N, with *N* = 11,976,628, where N is the total number of SNP markers used in this GWAS study). The x-axis shows the chromosome number for significant SNPs, while the y-axis shows all traits with significant SNP-trait associations. The width of the gray bar of a given chromosome number on the x-axis indicates the chromosomal position of that chromosome. The units of traits in y-axis; NUE (nitrogen use efficiency) is Kg Kg/m^2^; NRE at moderate and low N is %; dry biomass for low and moderate N is Kg; rectangles represent SNPs that are color coded based on *p*-value.

To uncover the genetic architecture of NUE, dry biomass at moderate N and dry biomass at low N, we explored the natural variation in the 331-switchgrass diversity panel genotyped with 11,976,627 SNP markers. The SNP numbers are after the minor allele frequency (MAF) filtration at 1%. For NRE at low N and NRE at moderate N, we explored the natural variation in 150 switchgrass accessions. We used the mixed linear model using GCTA (Yang et al., 2011) to perform the GWAS. At the specified threshold, overall, we obtained 19 unique (i.e., non-redundant) SNPs across Chromosome 2 (2K), 3 (3K), 4 (4K), 6 (6K), 7 (7K), 8 (8K), 13 (4N), 14 (5N), 15 (6N), and 18 (9N) (Figure 2 and Supplementary Table 5).

We identified 32 unique candidate genes from the 10-kb interval, as well as from the nearest left and right from each unique GWAS peak SNPs (Supplementary Table 5). The GWAS candidate gene list of NUE and the related traits in switchgrass, along with its Arabidopsis and rice orthologous gene description, can be found in Supplementary Table 5.

### Linkage Disequilibrium Analysis Supports Genome Wide Association Study Candidate Genes

We found the GWAS peak SNP Chr06N_45879490 (located in Chromosome 06N at 45879490 bp) being significantly associated with the dry biomass at moderate N (*p*-value, 5.43E-10), which led to two candidate genes: Pavir.6NG264600 and Pavir.6NG264700 (Supplementary Table 5). Gene Pavir.6NG264600 was found to be 7703 bp away from SNP Chr06N_45879490, while Pavir.6NG264700 was just 9 bp away from the SNP Chr06N_45879490 (Supplementary Table 5).

**FIGURE 3:**
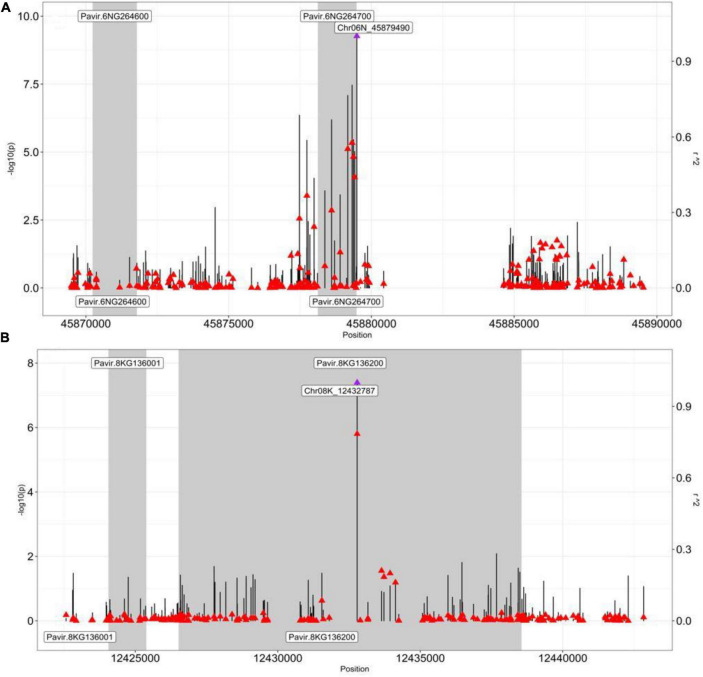
Pairwise LD analysis between the peak SNP and the SNP underlying genes. Pairwise LD estimates (*r2*) of the GWAS peak SNP with SNPs spanning a 20-kb (±10 kb) interval from the GWAS peak SNP. A scatterplot of the association results and LD estimates with the GWAS peak SNP **(A)** Chr06N_45879490 (dry biomass moderate N) and **(B)** Chr08K_12432787 (NUE), with SNPs spanning a 20-kb interval from their respective positions (bp). The negative log10-transformed *p*-values (left, y-axis) and *r*^2^ (right, y-axis) from the GWAS analysis are plotted against the genomic physical position. Vertical lines are the negative –log10 transformed *p*-values for individual SNPs from the GWAS results. Red triangles are pairwise LD *r*^2^ estimates of SNPs with the GWAS peak SNP (a purple triangle). Shaded bars designate genes. Gene Pavir.6NG264700 in panel **(A)** is *caffeoyl-CoA O-methyltransferase* (*CCoAOMT*), and gene Pavir.8KG136200 in panel **(B)** is *alfin-like 6* (*AL6*).

We also performed the pairwise LD analysis using the squared allele-frequency correlations (r^2^) between the GWAS peak SNP and the SNPs across 20 kb (10 kb up/downstream) of the peak SNPs. Emphasis was given between the GWAS peak SNPs and SNPs, residing within the candidate genes within the 20 kb region to better understand the LD association between them. Our LD analysis strongly supported the Pavir.6NG264700 to be a strong candidate gene for dry biomass at the moderate-N condition (Figure 3A). The peak SNP Chr06N_45879490 (Figure 3A—a purple arrow) was found to be in moderate to strong LD with the top 4 SNPs (Figure 3A—a red arrow, Supplementary Table 6), residing within gene Pavir.6NG264700, i.e., Chr06N _45879322 (r^2^, 0.57), Chr06N _45879172 (r^2^, 0.55), Chr06N _45879369 (r^2^, 0.52), Chr06N _45879412 (r^2^, 0.43) (Figure 3A and Supplementary Table 6). The SNPs residing within gene Pavir.6NG264600 had low LD (r^2^, 0.1), with the GWAS peak SNP Chr06N_45879490, suggesting gene Pavir.6NG264700 is worthwhile to explore for downstream analysis as compared to gene Pavir.6NG264600 (Figure 3A). We computed PVE for SNP Chr06N_45879490 and was found to be 0.104.

Next, we identified another interesting GWAS peak SNP Chr08K_12432787, which was significantly associated with the NUE (*p*-value, 4.07E-8) that led to the candidate gene Pavir.8KG136200 (Supplementary Table 5). Interestingly, the gene was found to be residing within the GWAS peak SNP Chr08K_12432787 (Figure 3B and Supplementary Table 5).

We again performed the pairwise LD analysis using the squared allele-frequency correlations (r^2^) between the GWAS peak SNPChr08K_12432787 and the SNPs residing within the candidate genes within the 20 kb region (10 kb up/down stream of GWAS peak SNP) to better understand the LD association between them. Our LD analysis strongly supported Pavir.8KG136200 to be a strong candidate gene for NUE (Figure 3B). The peak SNP Chr08K_12432787 (Figure 3B—a purple arrow) was found to be in strong LD with the SNPs Chr08K_12432786 (r^2^, 0.78) (Figure 3—a red arrow just below the purple arrow, Supplementary Table 7) residing within gene Pavir.8KG136200, supporting our candidate gene for NUE. On the other hand, GWAS peak SNPChr08K_12432787was found to be in low LD with SNP residing within gene Pavir.8KG136001 (Figure 3B and Supplementary Tables 5, 7). We also computed the PVE for SNP Chr08K_12432787 and was found to be 0.083.

## Discussion

Understanding the genetic basis of NUE is the key for developing nitrogen use-efficient switchgrass lines. To our knowledge, there is no documented study on the genetic basis of NUE in field-grown switchgrass at two contrasting nitrogen fertility treatments. Here, we studied the genetic basis of NUE in switchgrass. Our results provide interesting insights into the genetic architecture of NUE and its relationship among the various NUE-related traits. We highlighted *CCoAOMT* and *AL6* as important candidate genes to regulate NUE and the potential role of N treatments in modulating the expression of these genes.

### Response of N Fertilization to Switchgrass Accessions May Not Be Immediate and Requires Longer Establishment Time

We found the mean dry biomass for the switchgrass diversity panel grown in low- and moderate-N conditions to be similar (Table 1). One of the potential reasons to have a similar dry biomass mean for low- and moderate-N-grown switchgrass may be due to the early establishment of the switchgrass panel (Wolf and Fiske, 2009; Fike et al., 2017). The dry biomass data presented here were collected in the 2nd year of the switchgrass field trial. It has been reported that the time required to reach switchgrass full productivity can vary widely. The production guides often suggest that switchgrass stands may not be fully established (i.e., not fully productive) until the third growing season (Wolf and Fiske, 2009; Fike et al., 2017), which might be one of the reasons that the response to N fertilization could not be fully seen. Switchgrass productivity in response to N depends on several factors that include genotype; location; environmental conditions, such as precipitation and soil; and managements, such as harvest frequency and timing (Fike et al., 2017). Interestingly, our analysis on the mean N content at moderate N was found to be relatively higher than the mean N content at low N at both the mid-season and the end of season (Table 1). This indicates that the response to N treatment has started to show up but may need longer duration to significantly impact other phenotypic traits, such as dry biomass yield.

### Accessions Having Negative Nitrogen Use Efficiency Values Should Be Considered Acceptable, While Negative Nitrogen Remobilization Efficiency Values Should Be Considered Unacceptable for Breeding Switchgrass

We have found a range of both positive and negative values when calculating NUE and NRE in our study (Tables 1, 2). These positive and negative signs would play important roles in subsequent selection of switchgrass accessions for improving their NUEs. Positive NUE values of a switchgrass accession indicate the performance of the accession improved by the application of N fertility treatment as compared to the unfertilized condition (higher N responsiveness), and, hence, breeders could select these lines for the areas where there is ample abundance of N fertilizers (Han et al., 2015). However, care should be taken in applying the recommended dose of fertilizers to gain the benefit while, at the same time, mitigating the unwanted environmental consequences. Negative NUE values of a switchgrass accession indicate that the accession performs best even under low N and does not need to be supplemented with N fertilizers (high-genetic N efficiency) (Han et al., 2015). These accessions will be best to use from an NUE point of view and can be suitable to those areas with low-fertility soil status (Han et al., 2015). Hence, both categories of NUE could help breeders produce nitrogen use-efficient switchgrass accessions based on the choice of availability of N fertilization, response of accessions to N fertilizers, and with respect to protect the environment due to heavy use of N fertilizers. We identified the top 10 switchgrass accessions ranked based on the most positive and negative NUE values (Table 2); however, we want to emphasize that this result is based on a single location here at Knoxville, TN. Additional experiments are needed to evaluate these top accessions at different locations and at multiple time points to check the stability and consistency for both high-genetic N efficiency and high-N responsiveness. Therefore, these experiments warrant additional future evaluations. These selected accessions open the avenues to further breed for nitrogen use-efficient switchgrass accessions. It further explores the differential genes and the gene network to understand the underlying biological mechanism for the nitrogen use-efficient lines under contrasting N fertility conditions. Similar to NUE, we found NRE also had both positive and negative values in its distribution (Table 1). Positive NRE of a switchgrass accession indicates the N content in the tillers of the mid-season is higher than the N content in tillers at the end of season (Yang et al., 2016), which is an acceptable trait for breeding nitrogen remobilization efficiency. However, negative NRE values indicate N content in tillers of the end of season is higher than the N content in tillers at the mid-season, indicating these accessions are not favorable to breed further as they are inefficient to remobilize the N content (Yang et al., 2009). This information will be important for breeders to select the switchgrass accession that has the highest N content at the mid-season growth but lowest at the end-of-season growth.

### Gene Related to the Lignin Biosynthesis (*CCoAOMT*) Was Found to Be Associated With Nitrogen Use Efficiency-Related Traits

We found GWAS peak SNPChr06N_45879490 significantly associated with dry biomass at moderate N that led to candidate gene Pavir.6NG264700 (Supplementary Table 5). Pavir.6NG264700 was predicted to best hit the orthologous gene *caffeoyl-CoA O-methyltransferase* (*CCoAOMT*) in rice (LOC_Os08g38900) and S-adenosyl-L-methionine-dependent methyltransferases superfamily protein in Arabidopsis (AT4G34050) (Supplementary Table 5).

*CCoAOMT* is one of the key enzymes reported to be involved in the biosynthesis of monolignols (Wagner et al., 2011; Shen et al., 2013; Liu et al., 2016). In angiosperms, this enzyme is required for the biosynthesis of G- and S-type lignins (Meyermans et al., 2000; Wagner et al., 2011). Evidence has shown that the downregulation of *CCoAOMT* in *Pinus rediata*, *Medicago sativa*, and *Populustremula* × *Populus alba* leads to significant decrease of the G-type lignin but not the S-type lignin, inferring that *CCoAOMT* is mainly required for the biosynthesis of the G-type lignin (Zhong et al., 2000; Guo et al., 2001; Wagner et al., 2011; Liu et al., 2016). Previous studies in several species, such as maize (Li et al., 2013), pine (Wagner et al., 2011), and poplar (Lu et al., 2004), have reported that the suppression of *CCoAOMT* causes lignin reductions. Most of the *CCoAOMT* studies were conducted to understand its direct functional role; however, there are limited studies shown on the response of *CCoAOMT* on the environmental alternation, such as stress or change in plant or soil nutritional status.

A study conducted by Camargo et al. (2014) in *Eucalyptus* shows that the nitrogen fertilization could modulate the expression of *CCoAOMT* expression and its impact on the lignin and total biomass of the plant. Consequently, this would be important to improve the quality and composition of lignocellulosic feedstock, such as switchgrass. The study reported *Eucalyptus* (another bioenergy crop potential of producing lignocellulosic biofuels) grown at contrasting N treatments could be identified with significant differential expression levels of *CCoAOMT* between the two N contrasting treatments, supporting the dynamic role of N in regulating the expression of *CCoAOMT* (Camargo et al., 2014). Camargo et al. (2014) also reported that the expression of phenylpropanoid and lignin biosynthesis genes, such as *phenylalanine ammonia lyase (PAL)*, *Cinnamate-4-hydroxylase* (*C4H*), *4-coumarate CoA ligase* (*4CL*), *caffeic acid O-methyltransferase* (*COMT*), and *CCoAOMT* were downregulated in response to high N fertilization while upregulated in the N-limiting condition (Camargo et al., 2014).

*CCoAOMT* was mainly described as a key gene in the lignin biosynthesis pathway. Yet, with our GWAS result (Supplementary Table 5) and support from our LD analysis (Figure 3A and Supplementary Table 6), as well as from a previous study (Camargo et al., 2014), we propose *CCoAOMT* as a strong candidate gene for NUE in switchgrass. It is possible that N fertilization could affect the composition and quality of switchgrass lignocellulosic feedstock by modulating the expression of a lignin biosynthesis gene, such as *CCoAOMT* (Camargo et al., 2014). The future study should consider investigating the gene expression study of the *CCoAOMT* at two different N fertilization conditions in switchgrass.

### *AL6*-a Transcription Factor and a Strong Candidate for Nitrogen Use Efficiency in Switchgrass

Genome wide association study peak SNP Chr08K_12432787, which was significantly associated with the NUE, led to the candidate gene Pavir.8KG136200 and was predicted to best hit the orthologous PHD finger protein in rice (LOC_Os11g14010) and *AL6* in Arabidopsis (AT2G02470) (Supplementary Table 5). A limited study is found in the literature for the functional analysis of *AL6*. However, one study showed that *AL6* is involved in the root hair elongation during phosphate deficiency in Arabidopsis (Chandrika et al., 2013). The study identified a T-DNA mutant line from a large-scale genetic screen and found that it has a defect in root hair elongation, specifically under the low-phosphate condition. It was also shown that the mutant phenotype was caused by a mutation in the homeodomain protein *AL6* (Chandrika et al., 2013). The study further concluded that the *AL6* controls the transcription of a suite of genes (*ETC1*, *NPC4*, *SQD2*, and *PS2*); all of which are critical to root hair elongation (Chandrika et al., 2013). Up to now, to our knowledge, there has been no report on the possible role of the *AL6* gene in the N-deficient condition in the literature.

Since we identified *AL6* as a candidate gene associated for the NUE trait (Supplementary Table 5) and this gene is also supported by LD analysis (Figure 3B and Supplementary Table 7), our hypothesis is that, like the phosphate-deficiency condition, *AL6* may also regulate root hair elongation genes in the N-deficient condition. Hence, the variable expression of *AL6* in different switchgrass lines at different N conditions might be the reason for the natural variation of nitrogen use-efficient lines in our diversity GWAS panel. The roles of root hair elongation and growth are critical to nutrient absorption, uptake, and utilization and would be strong factors to contribute directly to the NUE. Gaudin et al. (2011) reported a decrease in root hair length under N stress in maize (Gaudin et al., 2011), while Foehse and Jungk (1983) found that tomato, rape, and spinach significantly increase root hair length when the nitrate concentration was decreased from 1,000 to 2 mM (Foehse and Jungk, 1983), suggesting the response to root hair in response to N availability may be species specific. For a future study, we will be exploring the functional role of *AL6* in switchgrass root hair in response to two N treatments, low and moderate. We will also perform the differential gene expression analysis of *AL6* at low and moderate N conditions using RNAseq analysis. It would be interesting to observe either of our hypothesis is supported or rejected.

## Conclusion

We have shown here that, by targeting NUE using a holistic approach, we can dissect its genetic architecture to identify novel SNPs and genes. We found genes related to lignin biosynthesis (*CCoAOMT)* and gene encoding root hair elongation (*AL6*), regulating the natural variation of NUE and related traits in switchgrass. We also highlighted the N fertilizer application potentially plays a role in modulating not only the biomass quantity but, more importantly, the biomass quality and composition. We identified the top ten nitrogen-efficient switchgrass accessions and uncovered the relationship among the various NUE-related traits. Our findings provide exciting possibilities to explore the underlying biological mechanism of NUE and to use marker-assisted selection and GWAS-assisted genomic selection in developing nitrogen use-efficient switchgrass cultivars.

## Data Availability Statement

The original contributions presented in the study are included in the article/Supplementary Material, further inquiries can be directed to the corresponding authors.

## Author Contributions

VS performed the experiments, processed and analyzed the data, and wrote the manuscript. HC performed the BLUP and GWAS analysis and assisted in writing the methodology. DK performed GWAS model optimization. YX assisted in writing and revisions to the manuscript. LH, CP, and BW participated in the field experiments and data collection. XW and MS performed nitrogen quantification analysis. DJ performed statistical and computational analysis. RM and MM designed the experiments, participated in result interpretation, supervised the work, and assisted in writing and revisions to the manuscript. CS conceived the study and its coordination, acquired funding, and assisted in interpretation of the results and the revisions to the manuscript. All authors contributed to the text and approved the final manuscript.

## Conflict of Interest

The authors declare that the research was conducted in the absence of any commercial or financial relationships that could be construed as a potential conflict of interest.

## Publisher’s Note

All claims expressed in this article are solely those of the authors and do not necessarily represent those of their affiliated organizations, or those of the publisher, the editors and the reviewers. Any product that may be evaluated in this article, or claim that may be made by its manufacturer, is not guaranteed or endorsed by the publisher.
